# Patient-reported characteristics of pernicious anaemia: a first step to initiate James Lind Alliance Priority Setting Partnership driven research

**DOI:** 10.1186/s12875-025-03036-0

**Published:** 2025-11-07

**Authors:** Alfie Thain, Petra Visser, Kathryn Hart, Ebba Nexo, Andrew McCaddon, Luciana Hannibal, Bruce HR Wolffenbuttel, Ralph Green, Nicola Ward, Catherine Heidi Seage, Katrina Burchell, Kourosh R. Ahmadi

**Affiliations:** 1 School of Biosciences and Medicine, University of Surrey, Guildford, UK; 2 The Pernicious Anaemia Society, Bridgend, UK; 3 Department of Clinical Biochemistry, Aarhus University Hospital, Aarhus, Denmark; 4 Faculty of Social and Life Sciences, Wrexham Glyndwr University, Wrexham, UK; 5 Laboratory of Clinical Biochemistry and Metabolism, Department of General Paediatrics, University of Freiburg, Freiburg, Germany; 6 Department of Endocrinology, University of Groningen, University Medical Center Groningen, Groningen, The Netherlands; 7 School of Medicine, University of California, Davis, CA USA; 8 Leicester School of Pharmacy, De Montfort University, Leicester, UK; 9 School of Sport and Health Sciences, Cardiff Metropolitan University, Cardiff, UK

**Keywords:** Pernicious anaemia, Vitamin B_12_ deficiency, Autoimmune disease, Diagnosis, Treatment

## Abstract

**Background:**

Pernicious anaemia (PA) is characterised by vitamin B_12_ deficiency due to autoimmune-mediated loss of gastric parietal cells and intrinsic factor - a specific transporter for B_12_’s intestinal uptake. The Pernicious Anaemia Society (PAS) is a patient-driven charity that recently identified 10 research priorities for improved diagnosis and management of PA through a James Lind Alliance Priority Setting Partnership. To facilitate research addressing these priorities, the aim of this study was to survey PAS members to identify and characterise a cohort of patients to form a PA research repository.

**Methods:**

An online survey was designed using SurveyMonkey (SurveyMonkey Inc., San Mateo, CA, USA). It comprised twenty-one questions collecting data on demographics, mode and timing of PA diagnosis, comorbidities, family history of PA or other autoimmune conditions, and type and regime of management. The survey was sent to 3,482 PAS members (April - September 2022) via the PAS website, newsletter, and email.

**Results:**

A total of 1,191 PAS members completed the survey. Of those individuals with a probable (*n* = 471) or suspected PA (*n* = 500) diagnosis defined by higher specificity diagnostics 84% were UK-based and 81% were female, with an age-range of 23–93 years. Diagnosis was predominantly based on low serum B_12_ (50%), positive intrinsic factor (38%), and/or parietal cell autoantibodies (15%). Diagnostic delays were common with 37% of participants reported waiting ≥ 3 years for a diagnosis. Nearly half of the participants suffered from one or more other autoimmune diseases. One-third also reported having at least 2 and up to 7 family members with PA or other autoimmune diseases. Vitamin B_12_ treatment frequency was highly varied, ranging from daily to 3 monthly B_12_ injections, with 52% of participants taking injections outside of the recommended guidelines.

**Conclusion:**

This study’s findings further highlight the gaps in current diagnostic and management approaches for PA and pave the way forward for future work in accordance with the JLA-PSP research priorities. By characterising a cohort of PA patients and compiling essential baseline data, we provide a foundation for research that supports the development of more effective diagnostic and management strategies.

**Supplementary Information:**

The online version contains supplementary material available at 10.1186/s12875-025-03036-0.

## Background

Pernicious anaemia (PA) is a multifactorial, life-long disorder resulting from an incompletely understood interplay of genetic, environmental, and autoimmune factors. The disease is characterised by vitamin B_12_ (B_12_) deficiency caused by B_12_ malabsorption as a consequence of autoimmune atrophic gastritis leading to reduced or absent intrinsic factor (IF) production [1–3]. Its prevalence is conservatively estimated at 0.1% in the general population, rising to 2% among those over 60 years in the UK [4, 5]. Frequently coexisting with other autoimmune disorders, PA contributes to a global rise in multimorbidity [6–9].

Management for PA usually involves life-long intramuscular injections of 1 mg hydroxocobalamin every 2–3 months [10]. However, this dosing regimen lacks a robust scientific basis, and many patients require more frequent injections to manage their symptoms adequately [11]. This has led to a number of patients self-managing their condition, including resorting to unofficial self-administration of intramuscular B_12_ injections [11, 12].

Despite its significant health and economic impact on the patients and health-care system, PA remains largely neglected by the global research community. This is largely due to the scattered nature of its diagnosis and treatment, which navigates both primary and secondary care, involving multiple medical specialities, including haematology, gastroenterology, and neurology. The establishment of the Pernicious Anaemia Society (PAS) was a direct response to these ongoing challenges [13]. As the only non-profit organisation dedicated solely to the improvement of PA diagnosis and management, PAS supports over 8,500 members and has actively supported and facilitated new research on PA. Notably, an important survey conducted by Hooper et al. in 2014 among PAS members revealed extensive diagnostic delays and dissatisfaction with treatment approaches, highlighting the urgent need for a change in PA management [14]. A more recent cross-sectional study revealed that this problem persists across several UK regions [15]. In 2020, the PAS partnered with the James Lind Alliance (JLA) to identify top research priorities through a Priority Setting Partnership (PSP), highlighting the need for more targeted research into PA’s diagnosis, treatment, and management [16]. Recent guidelines from the National Institute for Health and Care Excellence (NICE) echo these priorities, emphasising the need for improved diagnostic and management protocols whilst maximising cost-effectiveness [17]. In the UK, the NICE is an independent body that sets evidence-based standards for diagnosis, treatment, and management across the National Health Service (NHS) by developing recommendations for practitioners and commissioners, focusing on promoting good health and preventing and treating ill health. Its guidance ensures access to effective care, including for conditions such as vitamin B_12_ deficiency (https://www.nice.org.uk/guidance/ng239).

Benefitting from its unique access to a large sample of PA patients, PAS has enabled the gathering of extensive patient data on diagnosis, treatment, and prognosis of PA. The current manuscript draws on the most recent PAS survey dataset. We conducted exploratory analyses aimed at further highlighting patient-reported diagnostic delays and barriers to optimal treatment and management. Furthermore, we aimed to identify a subset of PAS members willing to participate in future studies aimed at answering research questions identified by both the James Lind Alliance-Priority Setting Partnership and NICE.

## Methods

### Recruitment

The survey was posted on the PAS members page and sent to all “active” PAS members (3,482 members) via email, newsletter, and website to inform them of the opportunity to participate in future research projects. An active member is considered a member who has an email associated with their membership and has consented to receiving emails from PAS. Inclusion criteria were membership of the PAS and aged 18 or above.

### Survey design

An online survey was created by the PAS in 2021–2022 using Survey Monkey (SurveyMonkey Inc., San Mateo, CA, USA). It is completed by all new members of PAS. It comprised twenty-one questions aimed at collecting data on demographics, mode, and timing of PA diagnosis, diagnosed comorbidities, specifically autoimmune disease, family history of PA and/or other autoimmune conditions, and type/regime of treatment; the categories and details are provided in Supplementary Table 1.

Data reported in the current manuscript is based on a secondary analysis of responses collected between April 2022 and September 2023, with all collected data transferred and stored in the PAS electronic database. Response options included yes/no, single selection, multi-tick, Likert scale or free text answers. Data from the survey was based solely on self-reported information and not confirmed by a medical examination. Each submission was checked for completeness and adherence to the survey’s requirements, with a minimum of 75% completion defined as the threshold for inclusion.

Participants were subsequently classified into the following diagnostic categories:

- Probable PA diagnosis – those diagnosed with high specificity diagnostics (a positive intrinsic factor autoantibody (IFA) result, gastroscopy or now obsolete Schilling test).
- Suspected PA diagnosis –a diagnosis of PA given by a health care professional (HCP) based on tests of lower specificity (parietal cell autoantibodies (PCA), low serum B_12_ levels, or elevated methylmalonic acid).
- No PA diagnosis - participants without an official PA diagnosis or those uncertain of their diagnostic status.

### Statistical analysis

Descriptive and stratified analyses were performed to investigate the associations between demographic characteristics, diagnosis timing, comorbid conditions, family medical history, and management strategies of the participants. Descriptive comparisons were initially made across all three diagnostic groups. However, to ensure the accuracy and relevance of further analyses, only data from participants with a probable or suspected diagnosis of PA were included in stratified and regression analyses.

The time from the onset of symptoms to the formal diagnosis of PA was categorised into predefined intervals to investigate the impact of diagnostic delays. Analyses also explored the relationship between familial PA status and several clinical outcomes, including treatment frequency, age at diagnosis, and the burden of autoimmune diseases. Participants were categorised based on their autoimmune disease (AID) burden into no additional AIDs, one additional AID, or multiple additional AIDs. Participants were stratified into two groups: “potentially sporadic” (reporting no other family members diagnosed with PA) and “familial” (having other family members diagnosed with PA or other AID).

We conducted univariate and multivariate logistic regression analyses to explore predictors of treatment frequency (standard [every 2–3 months] vs. more frequent injections). Predictors included sex (coded as male or female), age (continuous), and age at diagnosis (continuous, used as a proxy for age of onset). Time with symptoms before diagnosis was included both as a categorical variable (≤ 1 year, 1–3 years, 3 + years) and as an ordered factor to test for a linear trend. Autoimmune comorbidity (yes/no), other comorbidities (yes/no), family history of PA (yes/no), family history of other autoimmune disease (yes/no), and presence of other micronutrient deficiencies (iron, folate, vitamin D; each coded as yes/no) were also included as binary predictors. Treatment satisfaction was excluded from the final adjusted model due to interpretation issues related to self-sourcing of injections and measurement specific to HCP-provided care.

All statistical analyses were performed using R statistical software (version 2023.12.0 + 369). All analyses were conducted with a significance threshold set at *p* < 0.05.

## Results

### Characteristics of participants

The survey link was sent to 3,482 active PAS members, of which 1,191 members (34%) responded. Among the participants, 971 (81%) reported having a diagnosis of PA, while 125 (10%) stated they did not have an official diagnosis, and 95 (8%) were uncertain.

**Fig. 1:**
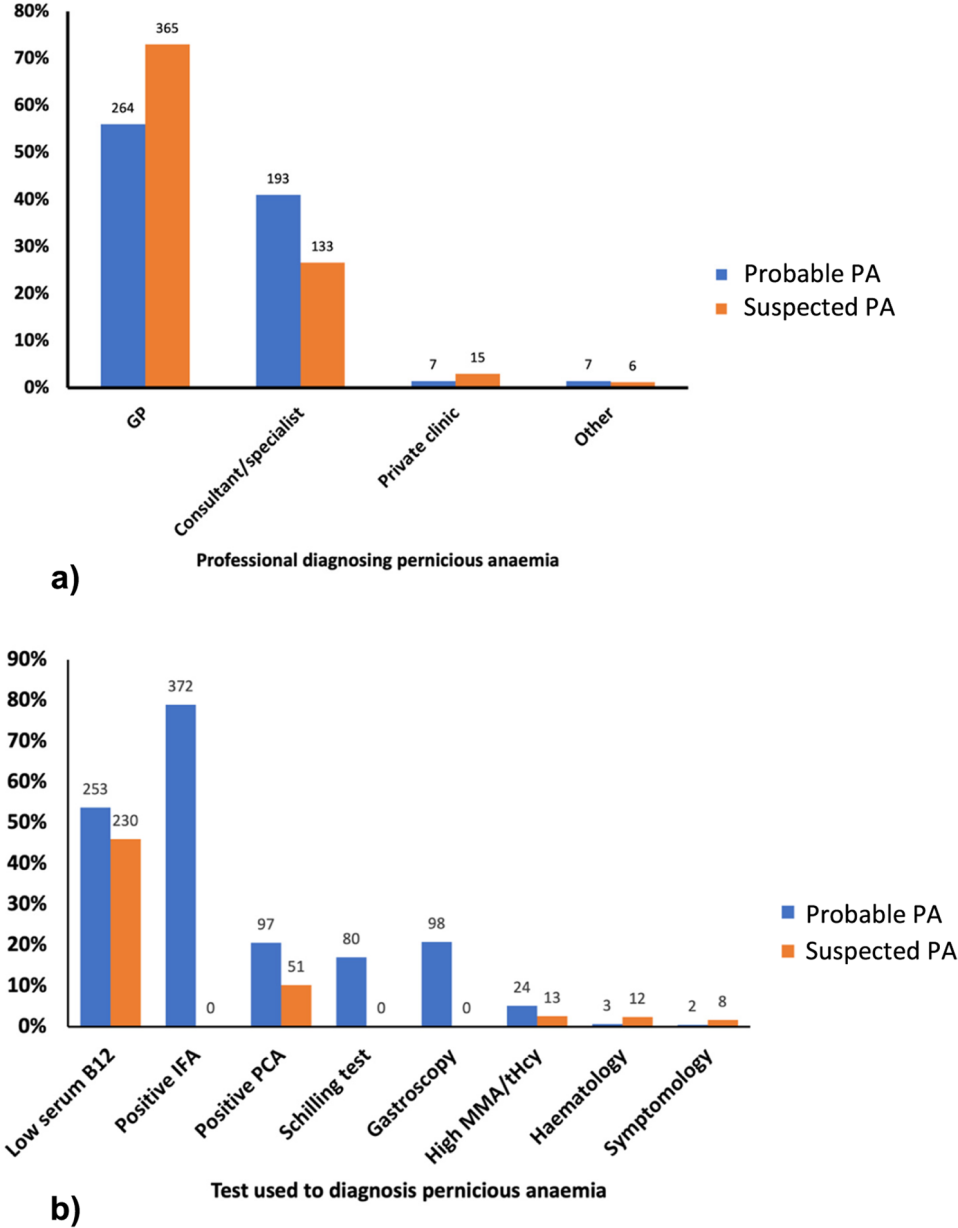
Diagnostic profiles reported by Pernicious Anaemia Society members. (**a**) Type of professionals diagnosing pernicious anaemia in the probable pernicious anaemia diagnosis group (n = 471) and suspected pernicious anaemia diagnosis group (n = 500) (**b**) Type of diagnostic tests used for pernicious anaemia in the probable pernicious anaemia diagnosis group (n = 471) and suspected pernicious anaemia group (n = 500). Haematology refers to individuals diagnosed through blood work, such as megaloblastic anaemia. Symptomology indicates that individuals are diagnosed based on clinical symptoms and/or a trial of B_12_ treatment in response to those symptoms

Participants were categorised into three diagnostic groups (Table 1). Those with probable PA (*n* = 471) were diagnosed with a positive IFA (*n* = 372, 79%), atrophic gastritis (*n* = 98, 20%), and/or a negative Schillings test (*n* = 80, 17%) (Fig. 1). The latter test is now obsolete and tested the patient’s ability to absorb B_12_ [18]. Patients grouped as suspected PA (*n* = 500) presented less stringent results, including a low plasma B_12_ (*n* = 230, 46%), a positive PCA (*n* = 51, 10%), high MMA (*n* = 13, 3%) or simply a diagnosis given by a HCP. Participants with no PA diagnosis (*n* = 220) showed none of the criteria listed for the first two groups (Supplementary Table 3).

**Table 1:** Diagnostic and sex-based comparative analysis of characteristics, diagnostics, comorbidities, and treatment trends in pernicious anaemia society members (*n* = 1191).

	Probable PA diagnosis^a^ (*n* = 471)			Suspected PA diagnosis^b^ (*n* = 500)			Combined PA diagnoses^c^ (*n* = 971)			No PA diagnosis^d^ (*n* = 220)		
	Female (%)	Male (%)	Total	Female (%)	Male (%)	Total (%)	Female (%)	Male (%)	Total (%)	Female (%)	Male (%)	Total (%)
Time with symptoms before diagnosis												
< 6 months	32 (8)	17 (21)	49 (11)	51 (14)	9 (9)	60 (13)	83 (11)	16 (9)	109 (11)	-	-	-
6 months − 1 year	75 (20)	16 (20)	91 (20)	84 (23)	29 (30)	114 (24)	159 (20)	45 (24)	205 (21)	-	-	-
1–3 years	112 (30)	21 (26)	134(29)	101 (27)	24 (24)	125 (27)	213 (27)	45 (24)	259 (27)	-	-	-
3–5 years	46 (12)	8 (10)	54 (12)	44 (12)	12 (12)	56 (12)	90 (11)	20 (11)	110 (11)	-	-	-
5 years +	113 (30)	20 (24)	133(29)	92 (25)	24 (24)	116 (25)	205 (26)	44 (24)	249 (26)	-	-	-
Vitamin B_12_ injection frequency												
Non-Standard												
More than weekly	21 (6)	6 (8)	27 (6)	30 (9)	6 (9)	37 (9)	51 (7)	12 (6)	64 (7)	23 (20)	7 (25)	30 (21)
Weekly	31 (9)	7 (9)	38 (9)	29 (9)	6 (7)	35 (8)	60 (8)	13 (7)	73 (8)	11 (10)	5 (18)	16 (11)
Every 2–3 weeks	35 (10)	11 (14)	46 (11)	16 (5)	7 (8)	23 (6)	51 (7)	18 (10)	69 (7)	8 (7)	4 (14)	12 (8)
Monthly	76 (22)	16 (21)	92 (22)	55 (17)	17 (20)	72 (17)	131 (17)	33 (18)	164 (17)	19 (17)	3 (11)	22 (15)
Standard												
Two monthly	82 (23)	12 (15)	94 (22)	80 (24)	15 (18)	95 (23)	162 (21)	27 (15)	189 (19)	22 (19)	3 (11)	25 (17)
Three monthly	105 (30)	26 (33)	131(31)	121 (37)	34 (40)	155 (37)	226 (29)	60 (32)	286 (29)	31 (27)	6 (21)	38 (27)
Additional Autoimmune diseases												
Hashimoto’s Disease	103 (27)	6 (7)	109(23)	78 (20)	16 (17)	94 (19)	181 (23)	22 (12)	203 (21)	28 (16)	2 (5)	30 (14)
Vitiligo	36 (9)	7 (9)	43 (9)	27 (7)	5 (5)	32 (6)	63 (8)	12 (6)	75 (8)	9 (5)	2 (5)	11 (5)
Rheumatoid Arthritis	28 (7)	3 (4)	31 (7)	25 (6)	14 (13)	39 (8)	53 (7)	17 (9)	70 (7)	8 (4)	3 (8)	11 (5)
Psoriasis	24 (6)	5 (6)	29 (6)	24 (6)	5 (5)	29 (6)	48 (6)	10 (5)	58 (6)	14 (8)	2 (5)	16 (7)
Graves’ Disease	17 (4)	3 (4)	20 (4)	18 (5)	1 (1)	19 (4)	35 (4)	4 (2)	39 (4)	7 (4)	0 (0)	7 (3)
Coeliac disease	14 (4)	2 (2)	16 (3)	8 (2)	0 (0)	8 (2)	22 (3)	3 (2)	24 (2)	2 (1)	0 (0)	2 (0)
Type 1 Diabetes	8 (2)	2 (2)	10 (2)	6 (2)	5 (5)	11 (2)	14 (2)	7 (4)	21 (2)	1 (1)	1 (3)	2 (1)
Any autoimmune disease*	183 (47)	21 (26)	204(43)	157 (40)	42 (40)	199 (40)	340 (43)	61 (33)	403 (42)	66 (37)	9 (23)	75 (34)
Other Comorbidities												
Asthma	128 (33)	16 (20)	144(31)	129 (33)	20 (19)	149 (30)	257 (33)	36 (19)	293 (30)	59 (33)	10 (25)	69 (31)
Depression	114 (29)	22 (27)	136(29)	104 (26)	18 (17)	122 (24)	218 (28)	40 (22)	258 (27)	40 (22)	12 (30)	52 (24)
Arrhythmia	53 (14)	11 (13)	64 (14)	31 (8)	12 (12)	43 (9)	84 (11)	23 (12)	107 (11)	23 (13)	6 (15)	29 (13)
Type 2 Diabetes	13 (3)	13 (16)	26 (6)	22 (6)	10 (10)	32 (6)	25 (3)	23 (12)	58 (6)	9 (5)	3 (8)	13 (6)
Family history of PA												
Familial PA	133 (34)	20 (24)	153(32)	131 (33)	30 (29)	161 (32)	264 (34)	50 (27)	314 (32)	50 (28)	9 (23)	60 (27)
Sporadic PA	225 (66)	62 (76)	318(68)	264 (67)	74 (71)	339 (68)	519 (66)	136(73)	657 (68)	129 (72)	31 (77)	160 (73)
Micronutrient deficiencies												
Iron	189 (49)	29 (35)	218(46)	153 (39)	21 (20)	174 (35)	342 (44)	50 (27)	392 (40)	54 (30)	9 (23)	64 (29)
Folate	101 (26)	22 (27)	123(26)	93 (24)	14 (13)	107 (21)	194 (25)	36 (19)	230 (24)	38 (21)	6 (15)	45 (20)
Vitamin D	125 (32)	24 (29)	149(32)	101 (26)	15 (14)	116 (23)	226 (29)	39 (21)	265 (27)	58 (32)	9 (23)	67 (30)
Contact for future research	438 (93)			453 (91)			891 (92)			162 (74)		

a, Probable PA Diagnosis: Includes participants diagnosed with high-specificity diagnostics, such as a positive Intrinsic Factor Antibody test, gastroscopy, or Schilling test (now obsolete)

b, Suspected PA Diagnosis: Includes participants with an official diagnosis from a healthcare professional based on tests of lower specificity, including parietal cell autoantibodies, low serum B_12_ levels, or elevated methylmalonic acid

c, Combined PA Diagnosis: Includes participants meeting Likely PA diagnosis and/or Possible PA diagnosis criteria

d, No PA Diagnosis: Includes participants without an official PA diagnosis or those uncertain of their diagnostic status.

*Other autoimmune diseases were reported including, Lichen Sclerosus, Sjogren’s syndrome, Addison’s Disease, Crohn’s Disease, Multiple Sclerosis

Results for each of those groups are presented in Table 1 and Supplementary Table 2. Initial descriptive comparisons were made across all three groups (probable, suspected, and no diagnosis). No significant differences were observed in diagnostic rates, autoimmune disease burden, or comorbidities. However, the probable PA group had higher rates of iron deficiency (46%) compared to the suspected PA (35%, *p* < 0.05) and the no diagnosis groups (29%, *p* < 0.05). The no PA diagnosis group had more participants having B_12_ injections more frequently than weekly (21%) compared to the probable PA (6%, *p* < 0.05) and suspected PA group (9%, *p* < 0.05). Given the similarity between the probable and suspected groups, further analysis focused on a ‘combined PA group’ (*n* = 971).

Of combined PA participants, 81% were female, 19% were male, and < 1% reported ‘prefer not to say’. The age range of the participants was 23–93, with 86% of participants aged 50 years old or greater (Supplementary Table 2).

### Diagnostic characteristics of participants

Age at diagnosis ranged from 10 to 80 years for combined PA and followed a normal distribution with a mean and median of 48 years.

The majority of combined PA participants were diagnosed within primary (e.g. general practice) (65%) or secondary care (e.g. hospital-based specialists such as haematology or gastroenterology) (34%) (Fig. 1). Approximately 37% (*n* = 359) of the participants reported waiting three years or longer with symptoms before receiving a diagnosis, whilst only 11% (*n* = 109) reported waiting less than 6 months (responses ranged from < 6 months to > 5 years) before receiving a diagnosis and starting treatment (Table 1). The average time since participants received their diagnosis was 14 years (SD = 10), with a range extending from less than a year to 55 years.

### Treatment regimens

The frequency of vitamin B_12_ injections in Table 1 illustrates the treatment frequency reported by PAS participants, ranging from daily injections to one injection every 3 months. The majority of B_12_ injections were in the form of hydroxocobalamin (70%), with other forms including cyanocobalamin (10%) and methylcobalamin (9%); Eleven per cent of participants were not sure of the injection form. Notably, 48% of participants had injections within the recommended treatment guidelines (2 or 3 monthly), while 52% reported having more frequent B_12_ injections than the guidelines. This deviation from the recommended guidelines encompassed a broad spectrum of injection frequencies, including monthly (17%), 2–3 weeks (7%), weekly (8%), and more than weekly (7%).

### Prevalence of comorbidities among participants

Among the combined PA participants, 35% reported one or more additional AID diagnoses. These ranged from a single AID diagnosis to up to 5 separate AID diagnoses. The most frequently reported AID comorbidities were Hashimoto’s disease (21%), vitiligo (8%), rheumatoid arthritis (7%), psoriasis (6%), Graves’ disease (4%), coeliac disease (3%) and type 1 diabetes (2%) (Table 1).

Approximately 67% of participants reported other non-AID comorbidities (Table 1), including asthma (30%), depression (27%), arrhythmia (11%) and type 2 diabetes (6%). In addition to B_12_ deficiency, 60% of respondents reported at least one or more other micronutrient deficiencies upon diagnosis of PA, most commonly deficiencies in iron (40%), folate (24%), and vitamin D (27%) (Table 1).

### Family history

Of combined PA participants, nearly one-third reported having at least one family member with PA, while 46% had at least one family member with another AID, and 19% had family members with both PA and other AIDs (Table 2). Generally, sisters, mothers and maternal grandmothers were the most commonly affected relatives. Additionally, 2% and 6% of participants reported having 3 or more family members with PA and AIDs, respectively (Supplementary Table 4).

**Table 2:** Family members with pernicious anaemia and autoimmune diseases reported by pernicious anaemia society members within the combined PA diagnosis group (*n* = 971)

	Family members with PA			Family members with other AIDs			Family members with PA + other AIDs		
	Female (%)	Male (%)	Total (%)	Female (%)	Male (%)	Total (%)	Female (%)	Male (%)	Total (%)
Mother	95 (12)	16 (9)	111 (11)	182 (23)	21 (11)	203 (21)	60 (8)	6 (3)	66 (7)
Father	44 (6)	12 (6)	56 (6)	83 (11)	17 (9)	100 (10)	32 (4)	5 (3)	37 (4)
Brother	26 (3)	2 (1)	28 (3)	58 (7)	12 (6)	70 (7)	18 (2)	2 (1)	20 (2)
Sister	51 (7)	11 (6)	62 (6)	109 (14)	11 (6)	120 (12)	35 (4)	4 (2)	39 (4)
Child	40 (5)	4 (2)	44 (5)	76 (10)	11 (6)	87 (9)	28 (4)	2 (1)	30 (3)
M. Grandfather	18 (2)	7 (4)	25 (3)	27 (3)	4 (2)	31 (3)	12 (2)	6 (3)	18 (2)
M. Grandmother	61 (8)	8 (4)	69 (7)	73 (9)	4 (2)	77 (8)	38 (4)	4 (2)	42 (4)
P. Grandfather	6 (1)	3 (2)	9 (1)	8 (1)	1 (1)	9 (1)	4 (1)	2 (1)	6 (1)
P. Grandmother	31 (4)	2 (1)	33 (3)	18 (2)	3 (2)	21 (2)	16 (2)	0 (1)	16 (2)
Any family member	264 (34)	50 (27)	314 (32)	383 (49)	63 (34)	446 (46)	161 (21)	24 (13)	185 (20)

Likely PA participants diagnosed with high-specificity diagnostics, such as a positive Intrinsic Factor Antibody test, gastroscopy, or Schilling test (now obsolete) and possible PA participants with an official diagnosis from a healthcare professional based on tests of lower specificity, including parietal cell autoantibodies, low serum B_12_ levels, or elevated methylmalonic acid; A single participant can be in multiple family member categories; Any family member indicates the number of participants with at least one family member in that grouping

*PA* Pernicious anaemia,* M* Maternal, *P* Paternal

### Familial versus sporadic cases of PA

Linear regression analysis was used to assess the relationship between familial PA status and the age at which individuals are diagnosed. The model estimated that individuals with familial PA are diagnosed, on average, 1.25 years earlier than those without familial PA, although this difference was not statistically significant (t= −1.35, *p* = 0.18).

As judged by logistic regression analysis, participants with familial PA were less likely to receive standard treatment compared to those with sporadic PA, although the result was not statistically significant (OR = 0.86, *p* = 0.28). Additionally, we assessed the association between treatment type (standard vs. non-standard) and family history of autoimmune diseases (including PA) versus no family history using a chi-square test. The results showed no significant association between these variables (*p* > 0.05). We also tested for the association between PA status (familial versus sporadic) and the burden of autoimmune diseases. Results revealed a highly significant association (x^2^ = 13.97, *p* < 0.01) between familial PA status and autoimmune disease burden. However, logistic regression analysis adjusted for sex, age, and age at diagnosis showed no significant association between familial PA (OR 1.09, *p* = 0.582) and increased likelihood of autoimmune diseases.

### Sex differences in outcome data

Comparative analysis between males and females indicated no significant difference in rates of PA diagnosis and frequency of B_12_ injections, as shown in Table 1. Amongst the combined diagnosis group there was a significantly higher prevalence of Hashimoto’s disease (23% vs. 12%, *p* < 0.05), any autoimmune disease (43% vs. 33%, *p* < 0.05), asthma (33% vs. 19%, *p* < 0.05), and iron deficiency (44% vs. 27%, *p* < 0.05) among females compared to males. Females were twice as likely to report having a maternal grandmother (8% vs. 4%, *p* < 0.05) with PA as males (Table 2). Females demonstrated a higher prevalence of relatives with AIDs than males, particularly mothers (23% vs. 11%, *p* < 0.05), sisters (14% vs. 6%), and maternal grandmothers (9% vs. 2%).

### Predictors of treatment frequency

We used univariate and multivariate analyses to identify which variables, individually or collectively, were associated with patterns of B_12_ injection frequency. Older age and time with symptoms before diagnosis were both significantly associated with injection frequency. Specifically, older age was associated with higher odds of receiving standard frequency treatment (OR = 1.03, 95% CI: 1.02–1.05, *p* < 0.001), indicating that younger patients were more likely to require more frequent injections.

As expected, the longer the patients exhibited symptoms before receiving a formal diagnosis, which ranged from under 6 months through to greater than 5 years, the more likely the patient was to be receiving injections more frequently than the standard 2–3 monthly. For example, participants who were symptomatic for 5 years or more had 54% lower odds of receiving standard treatment compared to those diagnosed within 6 months (OR = 0.46, 95% CI: 0.32–0.65, *p* < 0.001). A significant linear trend was also observed: each step increase in time symptomatic before diagnosis was associated with an approximate 50% reduction in the odds of receiving standard frequency injections (OR = 0.50, *p* < 0.001).

## Discussion

In response to the publication of ten research priorities related to the cause, diagnosis, management, and treatment of PA by the JLA-PSP, our study sought to establish a PA research repository. This repository is designed to promote and facilitate research that addresses these critical questions. Our study has begun to identify trends and associations that can help form hypotheses and guide future lines of research.

### A wide range of age-of-onset is reported for PA

Generally, for diseases, the younger the age of onset, the more important the role of common environmental and/or heritable factors. Conversely, the later the age of onset, the more prominent the unique environmental factors for the individual are [19]. We used ‘age of diagnosis’ as a rough proxy for ‘age-of-onset’ in participants and observed a near-normal distribution. The findings challenged the commonly held perception that PA primarily affects older adults [4].

### Exploring the aetiology of PA

Our study participants were predominantly female (81% with female to male ratio of 4:1). This reflects the known gender imbalance in PAS membership (85% female) [20] but also the higher prevalence of autoimmune conditions among females [14, 21–24].

We stratified our participants into two groups - sporadic and familial - hypothesising that different types of PA may exist. Our results suggest significant familial clustering and notably higher than rates (approximately 33% versus 20%) previously reported [25]. It is important to note that, whilst most participants identified no known familial link for their PA, this is likely partly due to a high rate of under/misdiagnosis associated with the condition [5]. The identification of families with multiple members with PA or other AIDs paves the way forward for future studies investigating the role of rare and common genetic variants associated with susceptibility and prognosis of PA. Furthermore, sporadic and familial forms of many other multifactorial diseases exist, and the observations reported here for PA are consistent with this [26, 27].

### Multimorbidity in PA participants

Multimorbidity, defined as the presence of two or more chronic conditions, was prevalent among the participants; half reported having one or more autoimmune diseases or other comorbid conditions [28]. Additionally, although not specifically detailed in this paper, cases of complex multimorbidity involving three or more conditions affecting at least three different body systems were observed. This distinction is crucial as the management of patients with PA alone should differ significantly from those with multiple chronic conditions, such as other AIDs. Appropriate management of these coexisting conditions is essential, as mismanagement could exacerbate symptomology, leading patients to believe they require more frequent B_12_ treatment when, in fact, better management of their other conditions might be needed.

### Diagnostic groupings

The diagnostic groupings were created and assessed to determine any impact of diagnosis method on presentation. Interestingly, no major differences were found between these groups, except for higher iron deficiency in the probable PA group. This suggests that, phenotypically, the individuals present similarly regardless of diagnostic criteria. Even in the ‘no diagnosis’ group, the presentation of the condition was broadly similar, in terms of demographics, comorbidities, and micronutrient deficiencies, suggesting that individuals in this group may in fact have undiagnosed PA. However, it is important to consider that not all participants may have PA. They may have B_12_ malabsorption due to other gastrointestinal issues, such as conditions like coeliac disease and Crohn’s disease or the use of medications like proton pump inhibitors [29]. Due to these diagnostic limitations, some individuals may be categorised as requiring B_12_ injections but not truly having PA.

### What might be causing diagnostic delays in PA?

Undoubtedly, as shown by our survey, obtaining a confirmed diagnosis of PA presents a significant challenge to patients. This is partly due to diagnostic challenges, including inadequate testing protocols and lack of a ‘gold standard’ for diagnosis. There is also a lack of awareness of PA among primary and secondary care practitioners [30–32]. Our survey highlights considerable variability in the diagnostic tests administered. Most participants were diagnosed solely through assessment of serum B_12_ levels, despite recognised concerns about the sensitivity and specificity of this test [33].

Due to the diagnostic limitations, a significant proportion of individuals in the ‘no PA diagnosis’ group likely have ‘undetected’ PA, given that most need regular B_12_ injections to relieve their symptoms. The absence of the Schilling test means that individuals are currently diagnosed either through assessment of serum B_12_, B_12_ metabolic markers (MMA or tHcy), or detection of IF or PC autoantibodies, but rarely with gastroscopic confirmation [18, 34].

The absence of a universally accepted ‘gold standard’ for accurately diagnosing PA poses a significant obstacle to optimising patient management and presents a substantial challenge for research in this field. While PCA and IFA tests are useful tools in the diagnostic workup of PA, both have important limitations. IFAs are highly specific (>95%) but have low sensitivity, being absent in over half of PA cases [35]. PCAs are more sensitive but less specific and may also be present in other autoimmune conditions [36, 37]. As such, PCA testing may be used as a screening tool to guide further investigations, including IFA testing. These limitations, alongside variability in testing practices across UK laboratories, underscore the diagnostic challenges in confirming PA through serological testing alone.

Several articles propose diagnostic criteria based on various tests for PA diagnosis but often lack specific or robust guidelines that describe a pathway or algorithm that clinicians can use to guide them [38, 39]. Other diagnostic algorithms to guide clinicians exist, yet they incorporate tests with poor sensitivity, such as the IFA test, or lack endoscopic testing [33, 40]. Furthermore, these algorithms often require the presence of objective parameters like anaemia, glossitis and a low serum B_12_, which will not be present in many true PA cases.

Since PA is defined by the advanced and end stages of autoimmune atrophic gastritis, gastroscopy, which allows for assessing gastric mucosal changes and sampling for diagnosing autoimmune atrophic gastric using the universal Sydney classification, is particularly important [41]. However, very few of our participants report being offered a gastroscopy. To support a more accurate PA diagnosis pipeline, gastroscopy should be routinely carried out with appropriate sampling techniques of the antral and corpus for histology testing, particularly in cases where there is clinical suspicion of PA but IFAs are negative [42]. Dottori et al. highlight that serum biomarkers such as pepsinogen, gastrin, PCA, and IFA can be used in combination as a ‘serum biopsy’ panel to help identify patients with high suspicion for autoimmune gastritis and PA who should be referred for gastroscopy with biopsies [39].

Timely diagnosis is critical in PA; the duration of symptoms influences symptom type, severity, and recovery [43]. Delaying B_12_ treatment for a prolonged period can result in irreversible neurological damage and permanent symptoms [38, 44, 45]. Vague and non-specific symptoms – due in part to autoimmune co-morbidities that present with similar symptoms - and limitations of tests, often exacerbated by lack of training and education [46], also contribute to misdiagnoses [31]. If there is a suspicion of PA but a diagnosis has not been reached, and other possible causes of the clinical presentation have been explored, it is important to trial B_12_ treatment and carefully monitor improvements in symptoms to assess the clinical picture.

Our findings further highlight that delays in patients receiving a diagnosis and commencing treatment play an important role in driving requirements for more frequent B_12_ injections to manage their symptoms effectively. Future guidelines and clinical practice are likely to benefit from incorporating this factor into decisions about treatment frequency, recognising that individuals with longer diagnostic delays may have greater symptom burden and treatment needs.

### A need for a more tailored vitamin B_12_ treatment regimen

The varied treatment frequencies observed strongly support the need for a more tailored approach to the management of PA, which goes beyond more frequent B_12_ injections [11]. The underlying basis for these different requirements remains unknown and warrants investigation. One clear finding is the widespread dissatisfaction among respondents, with many continuing to experience B_12_ deficiency-related symptoms when following current recommended guidelines [47]. It is important to highlight that the evidence to support current guidelines is poor, based on theoretical calculations on average B_12_ excretion in the urine [48].

Biomarkers of B_12_, including serum B_12_ and MMA, are of limited value in monitoring response or as a basis for prescribing a particular injection frequency [49]. Improved symptom monitoring and discovery of novel “response” biomarkers are, therefore, key targets for research. It will be critical to achieve tailored treatment regimens that lead to optimum symptom management and improved quality of life and prognosis for PA patients.

More than half of the participants reported receiving B_12_ injections outside of the recommended guidelines, which is likely under-reported. Anecdotal evidence suggests that recent treatment initiation, reluctance to self-inject, and financial constraints may all contribute to why more individuals are not seeking more frequent injections. A 2014 survey of PAS members reported that 13% of participants injected outside guidelines [14]. This perhaps indicates a potential shift in accessibility, knowledge and empowerment, or practitioner engagement. In the absence of objective PA biomarkers that correlate with patient symptoms, we support the principle that treatment decisions should be guided by patient-reported symptoms and signs assessed by the clinician, with individualised care prescribed based on clinical response and patient needs.

### Should we recognise sub-types of PA?

We have already highlighted that, from a purely genetic perspective, there may be at least two sub-types of PA - which we named sporadic and familial. We can also begin to formulate other ways to classify PA. From our survey and previous studies, we know that approximately 50% of PA patients present with other AID comorbidities, including vitiligo and autoimmune thyroid disease (Graves’ & Hashimoto’s), whilst in 50% of individuals, PA remains their only diagnosis.

The mechanisms of association between PA and other AIDs are unknown. The life course of PA allows stratification of the disease into those factors occurring prior to diagnosis (e.g., including *Helicobacter pylori* infection) or those associated as a consequence and life-course of the disease itself (i.e. iron, vitamin D or folate deficiency). Finally, we can also clearly describe sub-types of the disease based on treatment (type, mode) requirements. Follow-up studies using the proposed repository described here will aid the identification of other factors that could be used to stratify PA into different sub-types, contributing towards a new ‘precision medicine’ approach to future PA diagnosis and management.

### Proposed objectives of the PAS research repository

The primary objective is to support research aligned with JLA PSP priorities in PA and recent NICE guidelines [17]. Additionally, we aim to enhance participant recruitment through strategies developed with healthcare institutions and patient advocacy groups, focusing on integrating datasets from diverse sources into current and future studies. We also plan to facilitate medical records review and harmonise metadata from established biobanks, working with healthcare providers to securely access detailed medical histories, diagnoses, and treatment information. Finally, we will establish protocols for the collection, processing, and storage of biological samples (blood, urine, DNA/RNA), ensuring informed consent and addressing ethical considerations in collaboration with healthcare facilities.

New research to improve our understanding of the range of symptoms experienced by individuals with PA and their varying responses to treatment is urgently needed. The development of objective biomarkers to assess treatment response and to better understand why some individuals require more frequent B_12_ injections could address up to 7 out of the 10 questions of the JLA-PSP. The exploration of multi-omics-based technologies, including metabolomics and proteomics, the utility of wearables (smart-watches), and neurological tracking markers as tools to identify novel markers that objectively assess symptomology and response to treatment in PA, should be considered as priority aims [50, 51].

### Guidelines for researchers wanting to use the repository to tackle JLA questions

To facilitate research inquiries and collaborations utilising the PA Research Repository, we are in the process of forming an executive committee, comprised of representatives from the PAS committee, cluB-12 committee, researchers from the University of Surrey, and patients. Researchers interested in accessing the repository for studies aligned with the JLA research priorities or other related topics are encouraged to initiate contact through the corresponding author of this manuscript in the first instance.

### Strengths and limitations

The PAS research repository offers a unique resource with the potential to facilitate research, advance knowledge, and improve patient quality of life. The screening survey has provided valuable initial data and identified a willing cohort for future studies. However, there are several limitations to consider.

Firstly, the data is self-reported. While members of this patient group are highly engaged and knowledgeable about their condition, there is still the potential to introduce a degree of recall bias. Furthermore, participants were exclusively recruited from PAS, which will also introduce selection bias, making the results not fully representative of the broader PA population. This selective recruitment could indicate a higher motivation to manage their condition, potentially reflecting a more severe disease profile, dissatisfaction, or higher socioeconomic and educational status [52]. While the participant group is geographically representative of the UK (participants are predominantly UK-based), negative experiences may bias the dataset. However, this cohort includes individuals reporting high satisfaction with their treatment regimen despite their involvement and willingness to participate in research.

Some predictors in the regression analysis must be interpreted cautiously. Age at diagnosis was used as a rough proxy for age of onset, given the lack of a gold standard for diagnosis and the absence of a biomarker to determine onset. However, we recognise this will overestimate the true age of onset due to diagnostic delays. Similarly, treatment satisfaction was measured only in relation to healthcare professional–provided care; individuals who self-source injections may appear less satisfied with official NHS provision but remain satisfied with their overall symptom management.

Additionally, the absence of an independent, clinician-diagnosed group as well as a non-PA control group limits our ability to contextualise the prevalence of co-occurring conditions such as vitamin deficiencies or other comorbidities. Future studies by us and others should explore and validate our findings using appropriate comparison cohorts. More detailed diagnostic test results are also needed, particularly for individuals without a formal diagnosis. We also currently lack access to participants’ medical records, which limits our ability to validate diagnoses or explore diagnostic test results in detail. Future research will prioritise linkage with primary care data and direct recruitment from healthcare settings, allowing for validation of self-reported outcomes and further investigation of diagnostic pathways and treatment efficacy. We aim to further investigate and characterise individuals without a confirmed diagnosis, as their results suggest they underwent insufficient testing. This work will contribute to a better understanding of PA diagnosis.

## Conclusion

Our survey of PAS members has established the first ever PA research repository of over 1,000 patient participants and paved the way to address key questions outlined by the JLA-PSP and 2024 NICE Guidelines. An overview of the baseline data has given initial insight into the complexities of research into PA, from varied age-of-onset and familial clustering to diagnostic challenges and treatment variability. The potential complexity of PA, shown by the existence of distinct forms with varied aetiologies and manifestations, highlights the need for personalised screening and clinical care tailored to the specific form of PA or its presentation. This would improve diagnosis and ensure that management plans are optimally aligned with each patient’s unique health profile, thereby advancing towards a more precision-driven model in PA diagnosis and management.

## Supplementary Information

Supplementary Material 1.

## Data Availability

The datasets generated and/or analysed during the current study are not publicly available as the consent provided by participants does not include permission for public availability.

## Abbreviations

PA: Pernicious Anaemia
IF: Intrinsic Factor
PAS: Pernicious Anaemia Society
JLA: James Lind Alliance
AID: Autoimmune Disease
IFA: Intrinsic Factor Autoantibody
PCA: Parietal Cell Autoantibody
HCP: Health Care Professional
GP: General Practitioner
